# Neuropsychological profile of executive functions in autism spectrum disorder and schizophrenia spectrum disorders: a comparative group study in adults

**DOI:** 10.1007/s00406-022-01466-w

**Published:** 2022-09-05

**Authors:** Jo A. Yon-Hernández, Dominika Z. Wojcik, Laura García-García, María Magán-Maganto, Manuel Franco-Martín, Ricardo Canal-Bedia

**Affiliations:** 1 Universidad de Salamanca, InFoAutismo, Instituto Universitario de Integración en La Comunidad (INICO), Salamanca, Spain; 2 Zamora Hospital (Complejo Asistencial de Zamora), Zamora, Spain

**Keywords:** Autism spectrum disorder, Schizophrenia spectrum disorders, Executive functions, Task-based approach

## Abstract

As assessed by numerous neuropsychological tasks, individuals with autism spectrum disorder (ASD) and schizophrenia spectrum disorders (SSDs) have similar impairments related to executive functions (EFs). The neuropsychological profile of these two conditions was examined using the three-component EFs’ framework of Miyake and Friedman (Cogn Psychol 41(1):49-100, 2000). This approach assesses *Inhibition* (suppression of unwanted and irrelevant information/responses), *Updating* (use and control of contents of working memory), and *Shifting* (disengagement between activities or mental tasks) using nine different tasks. In line with previous research, we expected greater performance deficits in ASD in all three components compared to SSD, as well as faster responses for the SSD group. A self-paced task format allowed us to examine whether unlimited time given for a task would lead to better performance. The sample was constituted by the control group (*N* = 25), ASD group (*N* = 24), and SSD group (*N* = 12). Groups did not differ on *Inhibition* performance. In *Updating,* individuals with SSD performed poorer than the other groups. As for *Shifting*, both groups demonstrated poorer performance compared to controls, with the SSD group presenting the greatest difficulties. In terms of reaction time (RT), SSD participants’ RT were the slowest on *Inhibition* and *Shifting* tasks. There was a positive correlation between performance and time spent on *Inhibition* and *Shifting* only for the SSD group, which demonstrates that their performance improves when there are no time constraints. Our work provides a better understanding of spared and impaired EFs, which could be useful for designing strategies aimed at improving specific EFs in each group.

**Supplementary Information:**

The online version contains supplementary material available at 10.1007/s00406-022-01466-w.

## Introduction

Autism spectrum disorder (ASD) and schizophrenia spectrum disorder (SSD) are two conditions recognized by classification systems, such as the diagnostic and statistical manual of mental disorders—DSM-5 [1], as two distinct entities. In ASD, core symptoms include severe difficulties in social communication and restricted or repetitive patterns of behavior and interests. These symptoms usually manifest themselves in early childhood and are maintained throughout the individual’s life [1]. In SSD, however, symptoms usually appear in late adolescence or, more frequently, early adulthood. The symptoms of SSD are grouped into two categories: positive symptoms and negative symptoms [1]. Positive symptoms include hallucinations and/or delusions, whereas negative symptoms refer to the absence or reduction of behaviors such as emotional expression or the scarcity of communicative gestures.

In general, a person who displays typical clinical psychotic symptoms, such as hallucinations and delusions, would be diagnosed with SSD. However, many of the symptoms presented by people with ASD can be mistaken for SSD symptoms. For example, difficulties in social communication and social-emotional reciprocity, sensory disturbances, and rigidity in thinking are features shared by the two disorders [2–4]. Thus, the overlap in symptoms can lead to diagnostic confusion [4]. On a biological level, the two conditions share similar genetic modifications in DNA sequence (copy-number variations, CNV) and specific rare alleles [5]. On the neurological level, some magnetic resonance imaging (MRI) studies in ASD and SSD have shown similar alterations in the morphology of the posterior lobe of the cerebellum [6], and others demonstrated the same type of grey matter volume abnormalities in regions of the frontal and parietal lobes for the two conditions [7–9]. These neurobiological similarities, added to the similarities in behavioral symptomatology, generally make differential diagnosis difficult, especially in adulthood and if the individual did not receive a diagnosis of ASD in childhood. The present study aims to advance research on similarities and differences in the neuropsychological profiles of the two conditions. The long-term goal is to facilitate differential diagnosis and treatment strategies.

Executive Functions (EFs) are one of the aspects in which ASD and SSD present strikingly similar characteristics, which may influence the difficulties in differential diagnosis [10, 11]. EFs are known to be fundamental for learning, academic performance, mental health, adaptive behaviors [12, 13], and goal-directed behaviors [14]. Past research has demonstrated that the poor outcomes in personal, academic, vocational, or everyday functioning displayed by individuals with ASD or SSD have, indeed, been attributed to impairments in EF abilities [9]. Although scarce, the existing evidence suggests that, while in ASD the EF difficulties persist through adulthood [15], in SSD, there is a visible decline in EF in aging and after psychotic episodes [16].

Despite the lack of consensus on how to best assess EF, researchers agree that it is not a unitary domain but rather encapsulates a series of domains and abilities. Although there are different EF models, many of them suggest *Inhibition*, *Updating* and *Shifting* [12, 17] as the core components of EFs. This three-component conceptualization was first introduced by Miyake et al. [17] as the *Unity and Diversity* theory of executive functioning. The *Unity and Diversity* framework is a well-recognized and science-based assessment approach, wherein each EF component is measured using three different tasks. Miyake et al. [17] suggested that EF components work, both independently and interactively with one another. A confirmatory factor analysis (CFA) demonstrated that these three components are statistically separable into clusters, but since they are not perfectly correlated, they could still share a great portion of features between components [18]. Thus, the novelty of our study is to assess ASD and SSD EFs using Miyake and Friedman’s framework, which offers a task-based approach [17–20] that covers the core components of EFs found to be affected in the two disorders. We describe each component as follows.

### Inhibition

*Inhibition* is fundamental when it comes to suppressing unwanted responses to minimize the processing of irrelevant information and for selecting useful or relevant information to respond appropriately to a given situation [21], or to successfully complete a task [19, 22]. To date, literature on *Inhibition* in adults with ASD shows mixed results, with some studies showing spared functioning of this component [23], while others find impairments [24]. For example, in a study with adults with ASD, deficits were observed using a random-motor-generation task where participants were asked to inhibit motor-prepotent responses [25]. Individuals with SSD were also found to make more errors in inhibiting motor responses in tasks like the Stroop [21]. Furthermore, Ettinger et al., [21] showed that individuals with SSD make more mistakes when the task requires them to, not only inhibit a prepotent response, but also to produce an alternative response (e.g., in Antisaccade and Stroop tasks). However, in tasks where these individuals were required to only suppress an unwanted response (e.g., in the Stop-Signal task), their performance was intact.

### Updating

This component involves the ability to encode information in long-term memory, to retrieve it, and to subsequently use that information [12]. Moreover, it refers to the ability to monitor and control the contents of working memory and facilitates the access to relevant information [22]. In this fashion, *Updating* is very much linked to working memory capacity. Indeed, research suggests that for correct functioning, *Updating* requires working memory to incorporate new information of ongoing, planned behaviors and actions [12]. In their review, Gold et al. [26] found mixed results in SSD whereby some studies reported deficits and others report a spared *Updating* capacity. A great deal of research conducted in children and adolescents with ASD showed deficits in complex tasks that required both management of previous stored information and maintenance of immediate information, such as keeping track of stimuli. Furthermore, it seems that when the memory load is higher (e.g., keeping track of more than two objects), participants with ASD exhibit poorer performance [25]. A study assessing *Updating* in ASD and SSD revealed that both disorders have a lower working memory capacity as compared to healthy controls [11].

### Shifting

*Shifting* ability allows the individual to disengage from one activity or mental set to another. Also, it involves switching flexibly from one thought, action, activity, or situation to another [15, 22, 27]. Arguably, problems in *Shifting* can account for some repetitive and restrictive behaviors observed in ASD [28]. For example, Albein-Urios et al. [28] argued that 69% of young adults with ASD showed important difficulties in performing *Shifting* tasks as expressed by the Shift index of the BRIEF informant-report [29]. Sarro et al. [15] further suggested that *Shifting* difficulties are reflected in the fact that people with ASD persevere in their responses even after receiving corrective feedback on their performance. Similar impairments have been found in *Shifting* in SSD using neuropsychological tests like the Trail Making Test, showcasing similar perseverance and rigidity [30], as these individuals continued with the same response style after receiving negative feedback on their performance.

The Miyake and Friedman three-component framework [17, 31] is appropriate for our study given that it has been used in many clinical groups [8, 13], including SSD [22], and across different age groups (children [32] and adults [17, 19]). To our knowledge, our study is the first one that attempts to understand the pattern of shared and independent deficits in EFs in both ASD and SSD. The motivation behind carrying out a comparative study is twofold. First, it is highly relevant to be able to make a better differentiation of the two disorders, because the similarities in symptomatology can lead to misdiagnosis. Despite the fact that comparative studies that look for differences in underlying mechanisms that contribute to symptoms can be useful for correct diagnosis, the research is rather scarce. Thus, the present study responds to this scarcity of research. Second, the misdiagnosis can lead to inadequate treatment. Thus, in a long run, differentiating between spared and impaired aspects of EF in each disorder can improve treatment in terms of designing more personalized intervention programs.

Our work is the first to use a computerized Spanish language adaptation of Miyake and Friedman’s [17, 31] task-based assessment to compare individuals with ASD and SSD. Specifically, we were interested in examining performance accuracy and the average reaction﻿﻿﻿﻿-time (RT). Although the nature of our study is exploratory, a previous study comparing the performance of ASD and SSD on some neuropsychological tasks found that both groups showed a lower performance in *Inhibition-*, *Updating-*, and *Shifting*-related tasks [11]. However, it is noteworthy that the results published so far are mixed, possibly due to methodological issues (small sample sizes, variety of EF tasks used, etc.). Also, to be able to draw firmer conclusions about the status of EF similarities and differences, we need to directly compare SSD and ASD individuals’ performance on EF tasks. In line with past research, we expected to observe deficits in performance across all three-core EF components compared to the control group, but our predictions about the pattern of results are rather exploratory in nature. That is, we are interested in looking for specific differences in the pattern of strengths and weaknesses on performance scores in the two clinical groups. In terms of RT and bearing in mind previous findings, we expected to find faster responses in the SSD group compared to the ASD group [33]. We used a self-paced task format which allowed us to examine the relationship between time spent on a given task and performance accuracy. Research emphasizes the importance of considering RT because of its relevance when assessing EFs [33]. For example, a study which compared EF in individuals with ASD and ADHD found their performance to improve significantly in tasks that had no time limit [34]. Therefore, we expected to see a positive correlation whereby spending more time on a task would allow participants in our study to reach greater accuracy in their performance.

## Materials and methods

### Participants

All participants from this study were assessed with the Wechsler Adult Intelligence Scale-IV (WAIS-IV) [35]. The participants who scored below the cut-off point of ≥ 70 on the IQ-Full-Scale of the WAIS-IV were excluded from the study. Each group is described below.

*ASD group.* Twenty-four participants with ASD participated in the study. All participants met the IQ inclusion criteria. All participants were diagnosed with ASD prior the study; however, we also confirmed the ASD diagnosis with the Autism Diagnostic Observation Schedule (ADOS-2) (Modules 3–4) [36]. The reason behind confirming the diagnosis is that the majority of the participants had a diagnosis at an early age or did not have updated psychological records. Thus, to have a reliable characterization of the sample, we decided to confirm the clinical diagnosis using the ADOS-2. We were unable to confirm the diagnosis of two participants due to time constraints. However, since these participants had a previous official ASD diagnosis, we did not consider it should be of concern from a methodological perspective. Also, to assess autistic traits in all groups, we administered the Autism Spectrum Quotient Short Form Spanish version (AQ-S) [37]. The cut-off point for autistic traits is > 63 (see Table 1).

**Table 1:** Participants’ characteristics by the group

	Group			Kruskal–Wallis test					
	Mean (SD)			Group pairwise comparison					
	TDC (*n* = 25)	ASD (*n* = 24)	SSD (*n* = 12)	*H*	df	*p*	TDC-ASD	TDC-SSD	ASD-SSD
Age	28.48 (10.21)	29.38 (11.55)	42.75 (13.16)	9.68	2	.01	–	.010	.02
Age range	18–63	16–54	21–62						
FS-IQ	116.44 (16.72)	107.92 (20.50)	98.83 (17.84)	6.54	2	.04	–	.04	–
Verbal-IQ	131.60 (12.85)	121.83 (19.33)	119.83 (21.04)	4.09	2	.13	–	–	–
Performance-IQ	106.84 (18.85)	99.71 (22.68)	92.58 (21.18)	4.11	2	.13	–	–	–
AQ-S	51.16 (6.76)	75.96 (12.39)	63.58 (11.74)	37.06	2	.01	.01	.01	–

				*U*	*Z*	*p*			
ADOS-2		11.32 (2.84)	3.58 (3.92)	16.00	− 4.20	.01			
ADOS-COM		4.23 (1.19)	.83 (1.19)	7.00	− 4.60	.01			
ADOS-SI		7.09 (2.39)	2.58 (3.06)	35.00	− 3.52	.01			
ADOS-RRB		2.09 (1.34)	.67 (.78)	50.00	− 3.04	.01			
			(*n* = 11)						
PANSS-P			10.00 (2.79)						
PANSS-N			12.72 (5.46)						
PANSS-GP			24.27 (3.28)						
Psychopharmacological treatment									
Antipsychotic	0%	0%	100%						
Antidepressant	0%	8.3%	0%						
Anxiolytic	0%	4.3%	41.7%						
Mood stabilizer	0%	4.2%	16.7%						
Methylphenidate	0%	4.2%	0%						
Education level									
Mandatory school	0%	50%	58.3%						
University	100%	50%	41.7%						
Professional status									
Student	48%	50%	4.5%						
Employed	44%	12.5%	0.0%						
Unemployed	4%	37.5%	81.9%						
Retired	4%	0%	13.6%						

*TDC* Typical Developmental Controls, *ASD* Autism Spectrum Disorder, *SSD* Schizophrenia Spectrum Disorders, *FS-IQ* Full-Scale Intelligence Quotient, *IQ* Intelligence Quotient, *AQ-S* Autism Quotient Short, *ADOS* Autism Diagnostic Observation Schedule, *COM* Communication, *SI* Social Interaction, *RRB* Restricted and Repetitive Behavior. *H* Kruskal–Wallis *H* test, *U* Mann–Whitney *U* test, *DF* Degrees of Freedom, *Z* Z-Score Significance adjusted with Bonferroni correction *p* = .05

*SSD group.* Fifteen participants with a diagnosis of SSD participated in the study. According to the information provided by their psychiatrist, they had no history of substance abuse in the 5 years prior to the study (e.g., use of alcohol, cannabis, hallucinogens, or opioids). To participate, no acute psychotic symptoms could be present at the time of the assessment, as determined by the Positive and Negative Syndrome Scale (PANSS) Spanish version [38, 39] (see Table 1). To assess autism co-occurrence, the ADOS-2 was administered. Finally, three participants were excluded from the study as they scored below IQ-Full-Scale cut-off point.

*Typical Development Control group (TDC).* Twenty-five participants were recruited from the general public and student population from the University of Salamanca. All participants met the IQ inclusion criteria. The exclusion criterion for this group was a score above the cut-off point on the AQ-S. No participants were excluded.

A clinical questionnaire pertaining the use of medication, previous medical history, and other mental health problems was obtained from all participants. In the ASD group, one participant reported having epilepsy and four participants reported taking medication. As for the SSD group, antipsychotic medication doses were within the guidelines and doses recommended by Spanish drug regulators in all cases.

### Procedures

Before testing, informed consent for adult participants and parental consent for underage participants were collected. The study was approved by the Bioethical Committee of the Universidad de Salamanca. The testing was administered individually in two or three sessions, each with a maximum duration of 60–70 min. Sessions were conducted by a trained researcher.

### Neuropsychological tasks

We followed Miyake et al.’s [17] and Friedman et al.’s [31] procedures to assess EFs. The tasks were computerized using OpenSesame [40], a Python-based software. Tasks were administered in a MacBook Pro 13". As with the original study, *Updating* was examined with *Keep-Track, Letter-Memory* and *Spatial 2-Back task*. *Shifting* was assessed with *Number-Letter, Color-Shape,* and *Category-Switch task*. Finally, *Inhibition* was examined with *Antisaccade, Stop-Signal,* and the *Stroop task*. Specific information about the details and design of each task can be found on the electronic Supplementary Information document. For each of the nine tasks administered, we obtained individual scores that were later computed into an overall domain score for each EF component. For *Inhibition*, we calculated the Hit-Rate Performance score (HR-P, reflected by the total accurate responses) and the mean RT in milliseconds (MS). For the *Updating* component, we obtained the HR-P score, but no RTs were analyzed as in the original design [17]. As for the *Shifting* component, three different scores were obtained: HR-P, mean RT, and the Switch-Cost (SC), which was the average time participants took switching between tasks.

### Analysis

Analyses were conducted using SPSS 26.0 [41] and graphics were created using *R* [42]. We decided to run Kruskal–Wallis *H* test to examine group differences as well as post hoc analysis for all demographic characteristics. For the individual EF tasks, assumptions for conducting a parametric test were not met; therefore, we decided to run Kruskal–Wallis *H* test to determine group differences in the HR-P, RT, and SC across all tasks (see Table 2), as well as for the component total scores. Distributions of these variables were dissimilar for all groups, as assessed by visual inspection of a boxplot. Subsequently, pairwise group comparisons were performed using Dunn’s [43] procedure with a Bonferroni correction for multiple comparisons. Adjusted *p* values were reported with significance-level set at < 0.05.

**Table 2:** Tasks performance and reaction times by the groups

	Mean (SD)			Kruskal–Wallis *H* test				
	TDC	ASD	SSD	*H*	df	*p*	Group differences^a^	Pairwise group comparison^b^
Inhibition tasks HR-P								
	(*n* = 25)	(*n* = 24)	(*n* = 12)					
Antisaccade	1.43 (.15)	1.37 (.32)	1.31 (.35)	1.99	2	.37	–	–
	(*n* = 25)	(*n* = 24)	(*n* = 11)					
Stop-signal	1.12 (.35)	.93 (.32)	1.10 (.41)	3.39	2	.18	–	–
	(*n* = 25)	(*n* = 23)	(*n* = 9)					
Stroop	1.37 (.14)	1.19 (.35)	.96 (.36)	9.56	2	.01	TDC > ASD > SSD	TDC-SSD
Inhibition tasks RT								
	(*n* = 25)	(*n* = 24)	(*n* = 12)					
Antisaccade	393MS (157)	609MS (225)	831MS (327)	20.81	2	.01	TDC < ASD < SSD	TDC-ASD TDC-SSD
	(*n* = 25)	(*n* = 24)	(*n* = 11)					
Stop-signal	349MS (102)	512MS (272)	729MS (441)	11.86	2	.01	TDC < ASD < SSD	TDC-ASD TDC-SSD
	(*n* = 25)	(*n* = 23)	(*n* = 9)					
Stroop	922MS (173)	1259MS (496)	1327MS (220)	12.56	2	.01	TDC < ASD < SSD	TDC-ASD TDC-SSD
Updating tasks HR-P								
	(*n* = 25)	(*n* = 24)	(*n* = 9)					
Keep-track	1.18 (.22)	1.05 (.38)	1.02 (.29)	2.21	2	.33	–	–
Letter-memory	1.29 (.33)	1.12 (.39)	.92 (.30)	11.43	2	.01	TDC > ASD > SSD	TDC-ASD TDC-SSD
	(*n* = 25)	(*n* = 24)	(*n* = 10)					
Spatial 2-back	1.45 (.23)	1.38 (.25)	1.05 (.31)	12.59	2	.01	TDC > ASD > SSD	TDC-SSD ASD-SSD
Shifting tasks HR-P								
	(*n* = 25)	(*n* = 24)	(*n* = 9)					
Number-letter	1.42 (.162)	1.28 (.34)	1.08 (.28)	14.76	2	.01	TDC > ASD > SSD	TDC-ASD TDC-SSD
	(*n* = 25)	(*n* = 23)	(*n* = 10)					
Color-shape	1.38 (.19)	1.04 (.39)	1.04 (.53)	12.98	2	.01	TDC > SSD > ASD	TDC-ASD
	(*n* = 25)	(*n* = 24)	(*n* = 10)					
Category-switch	1.37 (.23)	1.14 (.35)	.77 (.21)	26.33	2	.01	TDC > ASD > SSD	TDC-ASD TDC-SSD ASD-SSD
Shifting tasks RT								
	(*n* = 25)	(*n* = 24)	(*n* = 9)					
Number-letter	1744MS (506)	2270MS (1166)	2844MS (1514)	5.91	2	.05	–	–
	(*n* = 25)	(*n* = 23)	(*n* = 10)					
Color-shape	1135MS (557)	1629MS (1110)	1755MS (677)	6.54	2	.04	TDC < ASD < SSD	TDC-SSD
	(*n* = 25)	(*n* = 24)	(*n* = 10)					
Category-switch	1336MS (355)	1882MS (1193)	1611MS (1030)	5.42	2	.07	–	–
Shifting tasks SC								
	(*n* = 25)	(*n* = 24)	(*n* = 9)					
Number-letter	712MS (507)	1186MS (1215)	1608MS (989)	9.34	2	.01	TCD < ASD < SSD	TDC-SSD
	(*n* = 25)	(*n* = 23)	(*n* = 10)					
Color-shape	781MS (587)	985MS (953)	1074.88MS (466)	4.22	2	.13	–	–
	(*n* = 25)	(*n* = 24)	(*n* = 10)					
Category-switch	593MS (308)	1066MS (972)	806MS (649)	3.10	2	.21	–	–

*SD* Standard deviation, *TDC* Typical developmental controls, *ASD* Autism spectrum disorder, *SSD* Schizophrenia spectrum disorders, *H* Kruskal-Wallis H test, *DF* Degrees of freedom, *HR-P* Hit-rate performance, *RT* Reaction-time, *MS* Milliseconds, *SC* Switch-cost

^a^Bonferroni correction for multiple comparison was used; statistical significance set at *p* = .05. Arrows showed the direction of the mean differences obtained from the groups

^b^Significant pairwise comparisons of the group. Statistical significance set at *p* = .05

Furthermore, we ran a Spearman’s correlation test to assess the relationship between accurate performance in the *Inhibition* and *Shifting* components and the RT from each group. Analyses showed that there were no outliers in our data and that the relationship found was linear with both variables normally distributed as assessed by the Shapiro–Wilk test (*p* > 0.05).

## Results

### Participants’ characteristics

Descriptive statistics of the participants’ characteristics are summarized in Table 1. We also reported differences between groups on some of those characteristics, as well as psychopharmacological use in Table 1.

### Tasks performance and RT analysis

**Fig. 1:**
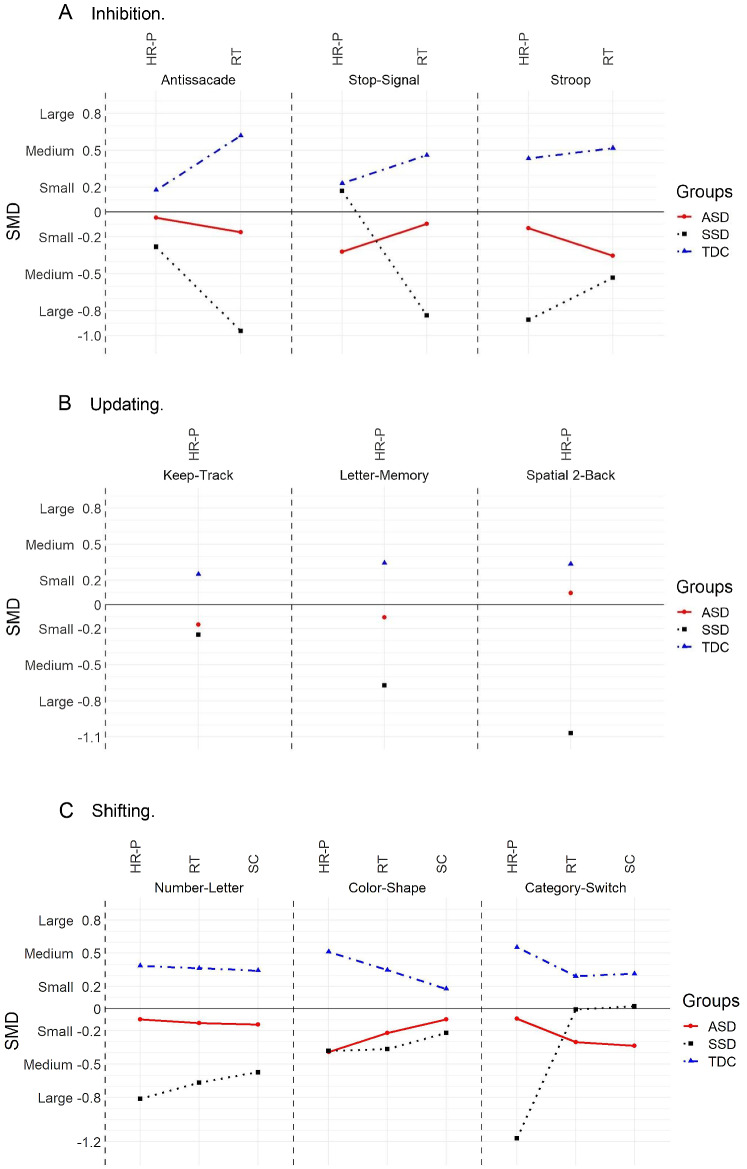
*SMD* standard mean difference, *HR-P* hit-rate performance, *RT* reaction-time, *ASD* autism spectrum disorder, *SSD* schizophrenia spectrum disorders, *TDC* typical developmental controls, *SC* switch-cost. **A** Inhibition tasks profile by the groups. **B** Updating tasks’ profile by the groups. **C** Shifting tasks’ profile by the groups. Group differences are described by the group effect size as small, medium, or large

The descriptive statistics of group performance in all the tasks are depicted in Table 2. One participant from the ASD group reported being colorblind, which made him unable to perform one task that relied on colors. Also, not every participant in the SSD group could complete all the tasks, mainly because they could not retain the instructions during the practice trials, and those tasks were not completed. The neuropsychological profile obtained in the tasks is shown in Fig. 1A, B and C. We plotted our results using standardized mean differences (SMD).

### Component performance and RT analysis

*Inhibition.* For this component, there were no significant group differences between the groups in their performance *H*(2) = 5.01, *p* = 0.08, *η*^2^ = 0.13, the group effect size was medium. As for the RT obtained in *Inhibition*, we found a significant group difference, *H*(2) = 26.70, *p* < 0.05, *η*^2^ = 0.33, and the group effect size was large. Post hoc analyses showed differences between the TDC and ASD and the TDC and SSD group. That is, SSD participants’ RT was the slowest (*M* = 973.06MS, SD = 214.74), followed by the ASD group (*M* = 782.87MS, SD = 262.51) and the TDC group (*M* = 555.18MS, SD = 100.20).

*Updating.* For this component, we found statistically significant group differences in performance, *H*(2) = 11.68, *p* < 0.05, *η*^2^ = 0.15, and effect size was large. Post hoc analysis only showed significant group differences between the TDC and SSD group, whereby participants with SSD performed poorer (*M* = 0.95, SD = 0.18) than the ASD (*M* = 1.12, SD = 0.28) and than TDC group (*M* = 1.23, SD = 0.17).

**Fig. 2:**
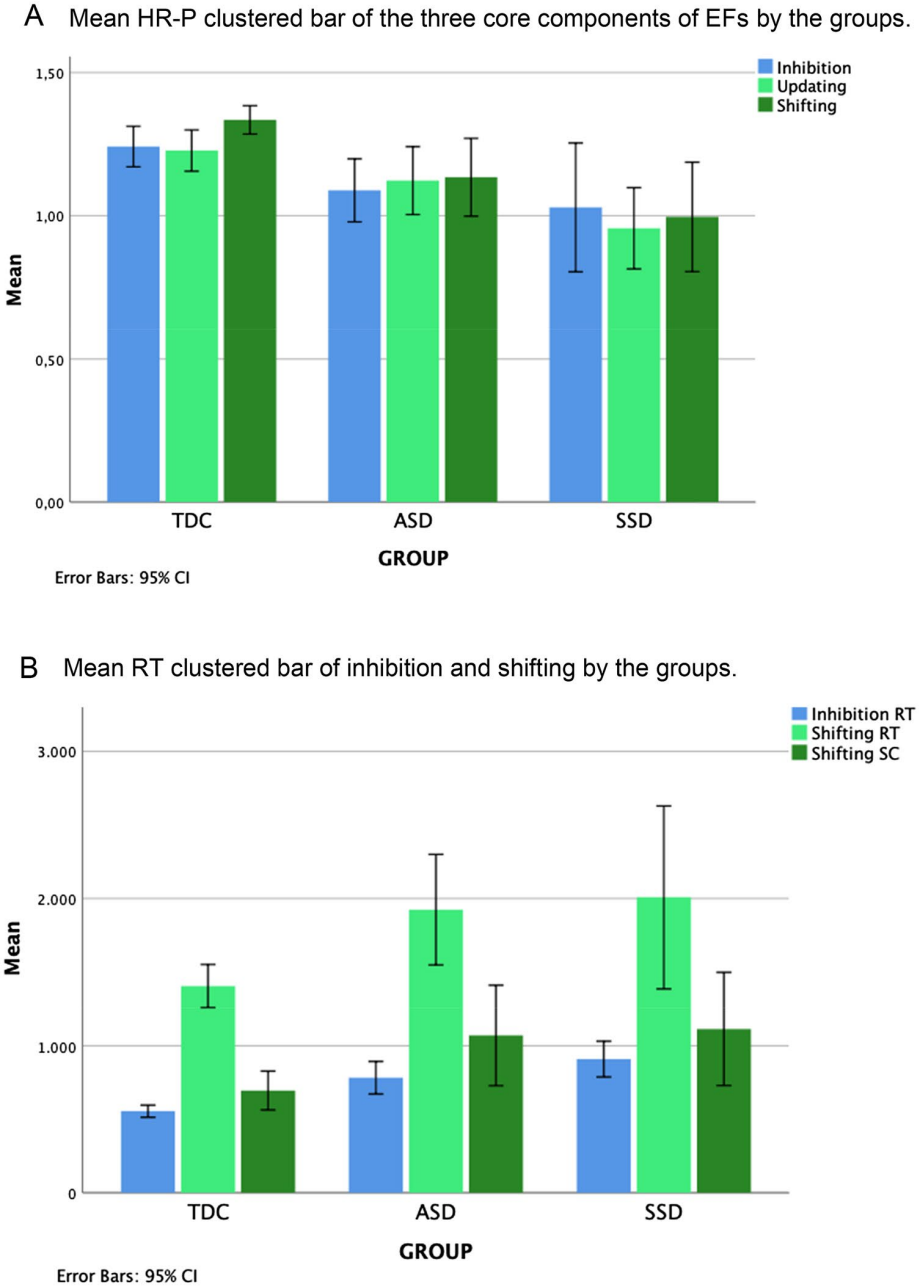
*HR-P* hit-rate performance, *EFs* executive functions, *RT* reaction-time, *ASD* autism spectrum disorder, *SSD* schizophrenia spectrum disorders, *TDC* typical developmental controls, *SC* switch-cost. **A** Mean HR-P scores in the three-core components of EFs by the groups. **B** Mean RT scores obtained in Inhibition and Shifting and the mean SC in Shifting by the groups

**Fig. 3:**
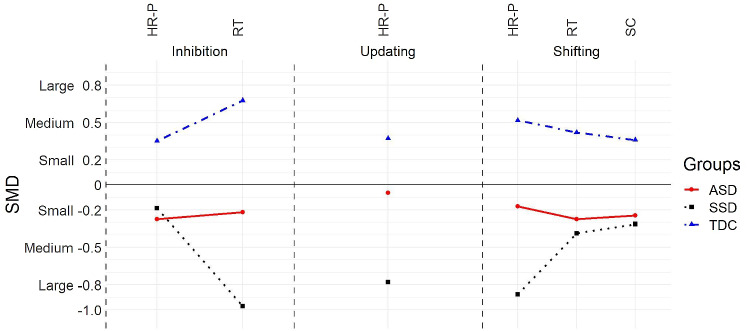
*SMD* standard mean difference, *HR-P* hit-rate performance, *RT* reaction-time, *ASD* Autism spectrum disorder, *SSD* Schizophrenia spectrum disorders, *TDC* Typical developmental controls, *SC* Switch-cost. Executive function profile in each core domain by the groups. Group differences are described by the group effect size as small, medium, or large

*Shifting.* Results indicated significant group differences in the performance of *Shifting H*(2) = 15.87, *p* < 0.05, *η*^2^ = 0.22, and the effect size was large. Post hoc showed a significant group difference in the performance between the TDC and ASD group and between the TDC and SSD group. Both SSD (*M* = 0.93, SD = 0.31) and ASD group (*M* = 1.13, SD = 0.32) performance accuracy was significantly lower than the TDC group (*M* = 1.34, SD = 0.12). As for the RT in *Shifting*, we found significant group differences *H*(2) = 7.26, *p* < 0.05, *η*^2^ = 0.15, with a large-effect size. Here, the SSD (*M* = 2008.13MS, SD = 867.80) and ASD (*M* = 1923.80MS, SD = 890.44) groups obtained the slowest responses compared to the TDC group (*M* = 1405.78MS, SD = 352.560). As for the Switch-Cost in *Shifting*, we did not find significant group differences, *H*(2) = 5.57, *p* = 0.06, *η*^2^ = 0.11 (see Fig. 2, A and B, for the average scores obtained by the groups). For visual representation of the neuropsychological profile in executive functioning by groups, see Fig. 3.

### Relationship between RT and performance accuracy

We found a strong positive correlation between the performance in *Inhibition* and time spent in the tasks (*r*_*s*_ = 0.58, *p* < 0.05) in the SSD group. The correlation was not significant for the TDC (*r*_*s*_ = 0.15, *p* = 0.49) and ASD groups (*r*_*s*_ =  − 0.15, *p* = 0.48). Similar results were found in *Shifting* performance score and the RT, where we found a strong positive correlation in the SSD group (*r*_*s*_ = 0.87, *p* < 0.05), but not the TDC (*r*_*s*_ =  − 0.35, *p* = 0.08) and ASD groups (*r*_*s*_ =  − 0.15, *p* = 0.49). Finally, the results demonstrated that the SSD group had the slowest RTs in *Inhibition* and *Shifting*.

## Discussion

The goal of this study was to examine the executive function profile of adults with ASD and SSD using, for the first time in the literature, a computerized task-based approach [17, 18]. Overall, we found that executive functioning difficulties were more pronounced in SSD than the ASD group. Also, contrary to what we expected, we found that the ASD group showed faster reaction times across the tasks compared to the SSD group.

When the *Inhibition* component was assessed, we found that all groups performed equally well, suggesting that the inhibitory mechanism was not altered in either of the groups. These findings show that, as assessed by tasks that target the suppression of irrelevant information or distractors, both ASD and SSD group’s performance was comparable to TDCs. However, individuals with SSD and ASD had slower RTs than controls, which indicates that they required significantly more time to complete these tasks as compared to the control group. When we looked at the relationship between RT and performance, only the SSD group showed the beneficial effect of having unlimited time—that is, the more time they spent on the task, the better their performance.

In terms of the *Updating* component, we found that individuals with ASD had comparable levels of performance to controls, contrary to what we predicted. Previous research in children and adolescents with ASD showed deficits in many aspects of the *Updating* component, such as problems with planning and monitoring actions; retrieving information from long-term memory; or updating ongoing activity [12, 22]. Given the results in our adult sample of individuals with ASD, it is plausible to think that this component of EF, while affected in childhood, does not remain impaired in adulthood [44]. As predicted, however, we observed poorer performance in SSD in *Updating* tasks compared to TDC performance. This finding is in line with previous studies in SSD that suggest great difficulties in *Updating* [16]. Note that reaction-time was not measured here.

With regards to the performance in the *Shifting* component, a clear pattern of difficulties in both individuals with ASD and with SSD was found, whereby they had significant difficulties in switching between activities accurately. Likewise, a different pattern of results from the TDC group was observed in terms of RT, in which the clinical groups took longer to complete the *Shifting* tasks. However, unlike with the inhibition tasks described above, more time spent on the task did not yield better performance levels in the ASD group. However, in the SSD group, there was a significant positive correlation between time and performance. A plausible explanation for this speed–accuracy trade-off in SSD is that participants with this disorder were older than the other comparison groups and slowing of reaction times with age is a common observation [45]. Notwithstanding, unlimited time did not bring the performance of individuals with SSD to typical levels observed in the control group. Finally, we did not find group differences in the Switch-Cost scores. This means that both groups were switching between tasks at a similar pace as the TDC group. It is worth noting, however, that although no significant differences were found between groups, some SSD participants did not complete parts of the tasks as they were unable to retain the instructions. This can be considered indicative of EF deficits whereby SSD individuals have problems in retaining information for the purpose of carrying out an ongoing task.

The outcomes from our study have several clinical implications. Assessing the strengths and weaknesses of EFs in these clinical groups may help us to establish not only the status of those aspects of the EF that are problematic, but also reinforce the use of those that are spared. For example, both individuals with SSD and ASD need support in tasks that involve *Updating* and *Shifting* skills, but not necessarily *Inhibition* skills. In terms of self-paced formats of activities, the outcomes of our study suggest that reducing time pressure is particularly beneficial for individuals with SSD, a finding that could be taken into account when planning vocational or occupational interventions. That is, a student with SSD could perform better in assignments by being given more time, while a professional with SSD could be provided with extended deadlines to aid their productivity. These specific interventions might influence the prognosis of achieving a successful adult life for individuals living with these disorders and essentially impact their quality of life, independence, and their ability to adapt to different day-to-day situations.

As mentioned earlier, a vast amount of research focuses on early development, and still little is known about EF abilities in later years. While *Updating* and *Inhibition* are typically impaired in childhood, they seem to be spared in adults with ASD. Some tentative accounts have been offered to explain the developmental trends in cognitive performance. A few studies in adults with ASD indicate that while certain EFs in autism are affected in early years, they improve with aging [46, 47]. Cognition and aging have received some attention in recent years. For example, Oberman and Pascual-Leone [48] found that older adults with autism do not present the same cognitive decline as older adults with early stages of Alzheimer Disease. This is particularly interesting, given that evidence shows memory and EF difficulties in children and adults with ASD. Oberman and Pascual-Leone [48] have explained this trend, suggesting that brain hyperplasticity in autism leads to brain underconnectivity in children and younger adults with ASD and contributes to cognitive impairments. However, during older age, it actually protects them from naturally occurring hypoplasticity in healthy aging (see literature on ASD [48, 49]). Also, the *safeguard hypothesis* [47] suggests that on a behavioral level, older adults with autism acquire, through life experiences, some compensatory strategies that help them to cope with their difficulties. Future longitudinal studies, or even cross-sectional studies with different age groups, could help us to ascertain the developmental trajectory of EFs in ASD and closely study the dynamic changes that seem to be occurring in EFs in this clinical group**.** On the other hand, the progression of cognitive trajectory in SSD is different to the one observed in ASD as there seems to be a decline, rather than an improvement in cognitive performance [50, 51].

### Limitations and future research

Given small sample sizes and given that this was an opportunity sample, we could not match the groups on age, making our outcomes hard to generalize for the SSD group. Therefore, we believe that future research should try to recruit larger samples and compare the groups considering age as a covariate, as age has been associated with poorer performance and slower responses in older individuals with schizophrenia [45]. Nevertheless, we should note that even though our sample size was small, we found medium-to-large-effect sizes when assessing group differences in performance across our study.

A recent work from Yon-Hernández et al. [52] found that ASD individuals self-report more difficulties than individuals with SSD related to both EFs and adaptive behaviors in everyday life situations. These results differ from the findings of the current study, where we noted relatively more EF problems in SSD than ASD population. To verify the extent to which EF deficits have an impact on these individuals' everyday functioning and ability to adapt in life, future research should focus more on combining both types of assessment, i.e., neuropsychological, and more ecologically valid evaluations of EF.

Research in children with ASD has associated difficulties in EFs with perseverative responses, stereotyped behaviors, and difficulties at modulating motor acts [44]. As these are shared symptoms in both autism and schizophrenia, future research should directly study the relationship between these behavioral manifestations and the core components of EFs. Also, there is some overlap in problems in social interaction in both disorders [53, 54]. Thus, it would be of interest to determine whether difficulties in interacting with other**s** are related to same (e.g., *Shifting*) or different (e.g., *Updating*) deficits in EF in these clinical populations.

## Supplementary Information

Below is the link to the electronic supplementary material.

Supplementary file1 (PDF 102 KB)

## Data Availability

The datasets used and/or analyzed during the current study are available from the corresponding author on reasonable request.
