# Supplementary material for: Neuropsychological profile of executive functions in autism spectrum disorder and schizophrenia spectrum disorders: a comparative group study in adults
Source: Eur Arch Psychiatry Clin Neurosci. 2022 Sep 5;273(3):719–30. doi: 10.1007/s00406-022-01466-w (PMC10085899; doi:10.1007/s00406-022-01466-w)
Supplement: Supplementary file 1 — Supplementary file1 (PDF 102 KB) [file 406_2022_1466_MOESM1_ESM.pdf]

Neuropsychological Profile of Executive Functions in Autism Spectrum Disorder and Schizophrenia Spectrum Disorders: A Comparison Group Study - Supplementary Information (SI)

Journal: European Archives of Psychiatry and Clinical Neuroscience

Authors: Jo A. Yon-Hernández[1], Dominika Z. Wojcik[1], Laura García-García[2], María Magán-Maganto[1], Ricardo Canal-Bedia[1], Manuel Franco-Martín[2]

Affiliation:

[1]Universidad de Salamanca – InFoAutismo – Instituto Universitario de Integración en la Comunidad (INICO).

[2]Zamora Hospital (Complejo Asistencial de Zamora).

Corresponding author: Dominika Z. Wojcik – d.z.wojcik@usal.es

Experimental Task Design

This study followed the original design for assessing Executive Functions (EFs) created by Miyake et al. [1] and Friedman et al. [2], wherein three different tasks were assigned to each component of *Inhibition*, *Updating* and *Shifting*, nine tasks in total. The stimuli/trials of each task were counterbalanced and randomized. Once randomized, the same order of stimuli/trials was used for all groups. Practice trials were included for each task to ensure that participants comprehended the instructions of the tasks. Each task was administered individually to avoid losing data due to external factors or cognitive fatigue. In this way, it was possible for the participant to take a short break in between tasks and resume them shortly after, or alternatively to schedule another session with the researcher. A pilot study was conducted to ensure there were no translation errors in the instructions and, consequently, that the stimuli remained clear.

Below, we explain the composition of each component.

Inhibition

This component included:

Antisaccade task adapted from Friedman et al. [2] and Roberts et al. [3]. Each trial began with a fixation cross appearing at the center of the screen for 1500 milliseconds (MS), followed by a yellow square introduced randomly on the left or right side of the screen. This was followed by the targeted stimuli, which was an arrow that stayed on the screen for 175MS on whichever side was opposite of the yellow square. Participants were instructed to not look at the square, but instead to make a rapid saccade to the opposite

side of where the arrow appeared. Following, participants pressed the correct direction key to indicate the direction the arrow pointed, e.g., if the arrow pointed to the left, they pressed the (←) key, if it pointed up (↑) or if it pointed to the right, the (→) key. After the practice trials, participants completed three blocks of 30 trials each.

Stop-Signal Task: adapted from Friedman et al., [2] and Verbruggen et al., [4-6], this task required participants to inhibit ongoing motor responses upon the presentation of a *Stop-Signal*. Each trial consisted of the appearance of a circle or a square on the screen, and participants were required to distinguish between the figures by pressing two different keys to indicate which figure was presented. If, during the task, participants heard a loud noise (*Stop-Signal*), they were instructed to stop. For the *Stop-Signal* trials the loud noise was randomly presented at 250MS or at 300MS after the stimuli presentation. We conducted three blocks of 32 trials each.

Stroop Task: Adapted from Friedman et al. [2] and Stroop [7]. This task involved selectively responding to the color of the word displayed while ignoring the actual meaning of the word. There were three different conditions for each trial: congruent (color and word matched), incongruent (color and word differ) and neutral trials (a colored square). A total of 130 trials were conducted for this task, 60 trials for the congruent condition, 60 for the incongruent condition, and 10 trials for the neutral condition. Different keys on the keyboard were assigned to each color; participants clicked the "*d*" key to indicate the color of the letter was red, the "*f*" key if it was green, the "*j*" key if it was black, and the "*k*" key if it was blue.

Updating

This component was assessed with:

Keep-Track, adapted from Friedman et al. [2] and Yntema & Schulman [8]. A list of six categories and their corresponding items were presented at the beginning of the task so participants could familiarize themselves with the categories and their respective items. The categories were animals, colors, countries, distances, metals, and relatives. At the beginning of each trial, participants were presented with the categories that they would need to keep track of during the trial. They were instructed to remember the last item that appeared on the screen from the indicated category. The sequence of each trial was as follows: first, 15 different words from all categories were presented randomly, one by one, at the center of the screen and for

1500MS each. Participants monitored all the words and had to remember the last word from the indicated categories by updating their memories constantly. There were three levels of difficulty. The more difficult had four categories, whereas the easier only had two. After the practice trials, 12 experimental trials were conducted, four of each difficulty level. Following each trial, participants submitted their responses in a text box.

Letter-Memory Task: Adapted from Friedman et al. [2] and Morris & Jones [9]. In this task, a series of letters were presented, one by one, in the center of the screen and for 2500MS per letter. Participants were then instructed to recall the last three letters of the sequence of each trial. To complete this task successfully, participants had to retain information and constantly update and modify the information they were receiving. The length of the lists presented were 5, 7, and 9 letters. Twelve trials were conducted, four of each difficulty. For example, if the list of 7 letters was "A, B, C, D, E, F, G," then the participants had to recall the following: "A…AB…ABC...BCD…CDE…DEF…EFG" and introduce the last three letters they recalled into the corresponding text box — in this case, "EFG."

Spatial 2-Back Task: Adapted from Friedman et al. [2] and Blacker et al. [10]. Participants were shown a black screen with white squares scattered in different locations. Their positioning remained the same until the end of the task. Each trial had a total duration of 1500MS, and each trial showed yellow squares flashing, one by one, for 500MS. One trial consisted of three stimuli flashes, to which participants indicated whether the first square that flashed was in the same position as the third square that flashed. They responded by pressing the spacebar. No response was required for non-targeted trials, i.e., the ones where the location from the first and third square did not match.

Shifting

The *Shifting* component was measured using three different tasks. Each one of them consisted of a block of 96 trials, in which 48 trials required that the individual switched between two tasks (*switching task trials*) and the other 48 trials did not (*no-switching task trials*). The same sequence presentation of the 96 trials was used for all participants. At the beginning of each trial, participants were given a cue indicating which task they had to perform. They were prompted to respond as quickly as possible by pressing the keys and with as few mistakes as possible.

In the *Number-Letter Task*, adapted from Friedman et al. [2] and Rogers & Monsell [11], the screen was divided into an upper and a lower section by a horizontal line at the center of the screen. A pair of characters, like a number-letter pair (e.g., 4L) or letter-number pair (e.g., L4), were presented in one of the sections. If the pair was presented on the upper section, participants were instructed to indicate if the number was odd (by pressing the "*f*" key) or even (by pressing the "*j*" key). If the pair of characters appeared at the lower section of the screen, they would have to indicate whether the letter was a vowel (by pressing the "*f*" key) or a consonant (by pressing the "*j*" key). To facilitate this task, the cue words "even" or "odd" and "vowel" or "consonant" appeared during the whole experiment.

In *Color-Shape Task*, adapted from Friedman et al. [2] and Miyake et al., [12], a red or green rectangle appeared at the center of the screen. Another figure, either a circle or a triangle, appeared inside the rectangle. At the beginning of the trial, a cue letter was presented at the top center of the screen. If the cue letter was "C," participants had to indicate the color of the rectangle. If the cue letter was an "S," they had to indicate what figure was inside the rectangle. They had to press the "*f*" key for red/circle and the "*j*" key for green/triangle.

Category-Switch Task, adapted from Friedman et al. [2] and Mayr & Kliegl [13], consisted of a list of 16 words (e.g., marble, bicycle, mushroom, shark, etc.). For each list, the participants had to categorize the word represented as (1) *living or non-living* or (2) if it was *smaller or larger than a soccer ball*. If a heart symbol appeared above the word, participants had to indicate whether the word represented *living vs. non-living:* for *living* they had to press the "*f*" key, and for *non-living* the "*j*" key. As for the other task, if an arrow-cross appeared above the word, they had to perform the *smaller or larger than a soccer ball task*. If it was *smaller*, they had to press the "*f*" key and if it was larger the "*j*" key.

References

1. Miyake A, Friedman NP, Emerson MJ, Witzki AH, Howerter A, Wager TD. The Unity and Diversity of Executive Functions and Their Contributions to Complex "Frontal Lobe" Tasks: A Latent Variable Analysis. Cognitive Psychology. 2000 Aug;41(1).

2. Friedman NP, Miyake A, Young SE, DeFries JC, Corley RP, Hewitt JK. Individual differences in executive functions are almost entirely genetic in origin. Journal of Experimental Psychology: General. 2008 May;137(2).

3. Roberts RJ, Hager LD, Heron C. Prefrontal cognitive processes: Working memory and inhibition in the antisaccade task. Journal of Experimental Psychology: General. 1994;123(4).

4. Verbruggen F, Logan GD. Models of response inhibition in the stop-signal and stop-change paradigms. Neuroscience & Biobehavioral Reviews. 2009 May;33(5).

5. Verbruggen F, Logan GD, Stevens MA. STOP-IT: Windows executable software for the stop-signal paradigm. Behavior Research Methods. 2008 May;40(2).

6. Verbruggen F, Logan GD. Response inhibition in the stop-signal paradigm. Trends in Cognitive Sciences. 2008 Nov;12(11).

7. Stroop JR. Studies of interference in serial verbal reactions. Journal of Experimental Psychology: General. 1992;121(1).

8. Yntema DB, Schulman GM. Response selection in keeping track of several things at once. Acta Psychologica. 1967;27.

9. Morris N, Jones DM. Memory updating in working memory: The role of the central executive. British Journal of Psychology. 1990 May;81(2).

10. Blacker KJ, Negoita S, Ewen JB, Courtney SM. N-back Versus Complex Span Working Memory Training. Journal of Cognitive Enhancement. 2017 Dec 16;1(4).

11. Rogers RD, Monsell S. Costs of a predictable switch between simple cognitive tasks. Journal of Experimental Psychology: General. 1995;124(2).

12. Miyake A, Emerson MJ, Padilla F, Ahn J. Inner speech as a retrieval aid for task goals: the effects of cue type and articulatory suppression in the random task cueing paradigm. Acta Psychologica. 2004 Feb;115(2–3).

13. Mayr U, Kliegl R. Task-set switching and long-term memory retrieval. Journal of Experimental Psychology: Learning, Memory, and Cognition. 2000;26(5).
